# University of Maryland, Dental Department—Annual Commencement for 1887

**Published:** 1887-03

**Authors:** 


### Editorial, Etc.

---

UNIVERSITY OF MARYLAND, DENTAL DEPARTMENT.—*Annual Commencement for 1887.*—The commencement exercises were held in the Academy of Music, Baltimore, on Wednesday, March 16th, 1887, in the presence of a large audience. The exercises opened with prayer by the Rev. Dr. Julius Grammer, of St. Peter's P. E. Church.

The Mandamus was read by the Dean, Prof. Ferdinand J. S. Gorgas, M. D., D. D. S. The degree of "Doctor of Dental Surgery," was conferred upon the following gentlemen, *fifty-one* in number, by the Hon. Severn Teackle Wallis, L. L. D., Provost of the University.

JULIUS ALBRECHT....................Germany
JOSEPH MABEN BAKER....................Arkansas
ALONZO AMASA BEMIS....................Massachusetts
DANIEL B. BLAUVELT....................New York
GARABED BOYAJIAN....................Asiatic Turkey
CHARLES J. BRAWNER....................Georgia
HENRY E. CHASE....................Massachusetts
JOHN G. CHISHOLM....................Alabama
FRED. JULIAN CROWELL....................New Hampshire
HENRY E. DOUGLASS....................New York
JOHN TRIPNER EIKER....................District of Columbia
GEORGE McCLENAHEN FAULKNER....................Pennsylvania
W. CRAWFORD YOUNG FERGUSON....................Canada
CHRISTIAN G. FRANTZ....................Pennsylvania
JOEL NELSON FURMAN....................New York

HEINRICH GARBRECHT............................Germany
WILLIAM F. GRAY.............................Virginia
L. LEE HARBAN...............................Maryland
EDWIN L. HARRIS........................Massachusetts
ANTON JOSEPH HECKER..........................Germany
SAMUEL W. HOOPES............................Maryland
HAMILTON V. HORTON.....................North Carolina
MICHAEL HOURIHANE...........................Virginia
MAX JAENICKE.................................Germany
PAUL JAENICKE................................Germany
JAMES LEITCH KEAN...........................Virginia
CHARLES T. LOVING..............................Texas
WILLIAM M. MEADOR, M. D.................South Carolina
SAMUEL McCOLL.................................Canada
JAMES H. A. MILLER.......................West Virginia
WELLINGTON C. MILLER.....................Pennsylvania
WOODSON N. MURPHY..............................Texas
JOHN H. NEILL.................................New York
ALBERTO LOPEZ DE OLIVEIRA.......................Brazil
HARRY HOMER PHILLIPS.....................Pennsylvania
PRESTON A. RAMBO..............................Georgia
SAMUEL S. REAMER.............................Virginia
WALTER FRANKLIN RICHARDS......................Illinois
WILFRED A. ROBERTSON............................Canada
CARY CLIFTON SAPP......................North Carolina
EDWARD H. SHIELDS................................Ohio
HENRY RUTGERS SHINE............................Florida
RICHARD ALEXANDER SHINE, JR....................Florida
E. EVERETT P. SLEPPY.....................Pennsylvania
PARKE P. STARKE..............................Virginia
ROBERT W. TALBOTT..................District of Columbia
MATHEW W. WHITE........................South Carolina
ARNOLD WIETFELDT...............................Germany
HEINRICH THEODOR WILHELM......................Germany
JOHN H. WILSON...............................New York
WILLIAM W. WOGAN.........................Pennsylvania

Mr. Wallis gave the graduates some very good, solid advice. He also awarded the prizes, as follows: University prize, for highest number of votes at final examinations, to M. Hourihane; honorable mention to W. C. Miller. S. S. White prize, for best two fillings of cohesive and non-cohesive gold foil to Preston A. Rambo; honorable mention to Henry F. Chase. Snowden & Cowman prize, for best set of continuous gum to H. H. Phillips; honorable mention to E. H. Shields. S. S.

White prize, for best partial set of teeth on metal, to M. Hourihane; honorable mention to L. L. Harban. Francis Arnold prize, for best full upper set of gum teeth on metal, to R. W. Talbott; honorable mention to W. F. Richards. Dr. Frank L. Wood prize, for best cohesive gold filling, to C. C. Sapp. Dr. James H. Harris prize, for best non-cohesive gold filling, to Hamilton V. Horton; honorable mention to D. B. Blauvelt. Dr. F. J. S. Gorgas prize, for best crown or bridgework, to Paul Jaenicke; honorable mention to Charles T. Loving. Dr. John C. Uhler prize, for best combination set on rubber or celluloid, with soldered rim, to Max Jaenicke, honorable mention to A. J. Hecker. Dr. Charles L. Steel prize, for best specimen tooth filling, to R. W. Talbott. Dr. B. H. Catching prize, for best examination in dental materia medica and therapeutics, to M. Hourihane, honorable mention to W. C. Miller.

An address was delivered by Rev. A. C. Dixon, D. D. He said: "I beg of you or a few minutes what you will be seeking all your lives, 'patience.' There is a close relation between dentistry and religion; it is a hard thing for ministers to give consolation to dyspeptics, neuralgics and toothachists, and we wish to turn all such people over to you. There is also a relation between dentistry and crime· it is said that when the east wind blows crime increases about 15 per cent., but when toothache is raging it increases about 40 per cent. It is also said there is a relation between dentistry and matrimony. A man comes home irritated with his business affairs and meets his wife, irritated with a bad case of jumping toothache, and then comes the quarrel from which the divorce court lawyers make money. If you have anything tender to say, don't say it to her who hath the toothache. Drunkenness is often brought on by the toothache, and so is the opium habit. People take whiskey and opium to kill the pain when their teeth are aching, and continue the use of these for fear their teeth may ache again. And now we invite you to become a member of the fraternity. If you believe in religion, temperance and matrimony, be good dentists. It will be your business to fill, and be sure to fill with the right sort of material, and in proportion to the knowledge that fills your heads.

Teeth come solid and become hollow. Heads come hollow and have to be filled. Acquire knowledge, and be wise enough to use it; but don't think you know more than you do. A wise fool is the worst sort of a fool. I recommend tender nerves and pulp for conscience, and enamel for will; but I know of some dentists who have wills of pulp and consciences of enamel. Have sound and permanent convictions; know what you will do and how to do it. Have faith in your profession. Faith is powerful, doubt is destructive."

The benediction was pronounced by Rev. Dr. Grammer, and the audience dismissed.

The number of Matriculates for the session of 1886-87 was *one hundred and twenty-four.*

The fifth annual banquet of the Alumni Association was held in the evening at the Howard House. Officers for the ensuing year were elected, as follows: Dr. R. S. Dodson, president; Dr. Elmer J. Wisherd, vice-president; Dr. J. S. Kloeber, secretary; Dr. J. R. Davis, treasurer. The annual address was delivered by Dr. F. J. S. Gorgas, dean of the school. About one hundred persons were present.

